# Utilization of lignocellulosic mushroom cultivation residues for bioethanol production via consolidated bioprocessing

**DOI:** 10.1007/s11274-026-05009-6

**Published:** 2026-05-12

**Authors:** Jeison Torzecki Bigolin, Jéssica Mulinari, Luciane Maria Colla

**Affiliations:** 1 Graduate Program in Civil and Environmental Engineering (PPGENG), University of Passo Fundo (UPF), RS Passo Fundo, Brazil; 2 Graduate Program in Agronomy (PPGAGRO), University of Passo Fundo (UPF), Campus I, L1 Building, BR 285, São José, Passo Fundo, RS 99052-900 Brazil

**Keywords:** Co-cultures, Lignocellulosic biomass, *Pleurotus ostreatus*, Pretreatment, Renewable energy, Second-generation bioethanol

## Abstract

In the context of the energy transition, there is an increasing need for research focused on low-cost feedstocks, such as lignocellulosic biomass, and on process optimization for the sustainable production of biofuels. Traditionally, processing lignocellulosic materials requires multiple stages. In this scenario, consolidated bioprocessing (CBP) emerges as a promising alternative by integrating enzyme production, hydrolysis, and fermentation into a single step. This study aimed to develop a CBP for bioethanol production using residues from oyster mushroom cultivation (*Pleurotus ostreatus*), composed of *Pinus elliottii* sawdust and soybean hulls (*Glycine max*). Preliminary tests were conducted to define the CBP conditions: the effects of biological (mushroom cultivation) and alkaline (sodium hydroxide) pretreatments were evaluated, and the production of cellulolytic enzymes by *Trichoderma* sp. was investigated in solid-state fermentation (SSF) and submerged fermentation (SmF) using the raw and pretreated residues. After selecting the optimal conditions, a 2³ experimental design was carried out to assess the influence of wheat bran supplementation, commercial enzyme addition, and co-culturing (*Saccharomyces cerevisiae* and *Bacillus velezensis*) on ethanol production. Cellulolytic enzyme production was observed in both fermentation types, with the SSF using only biologically pretreated substrate yielding the highest activity (2.04 ± 0.17 U/g). In CBP, the supplementation with commercial enzyme extracts, wheat bran addition, and co-culture showed positive effects on ethanol production, resulting in 7.07 ± 0.22 g/L, representing a 15-fold increase compared to the unenhanced process. These results highlight the potential of reusing mushroom cultivation residues for sustainable bioethanol production through CBP.

**Graphical Abstract:**

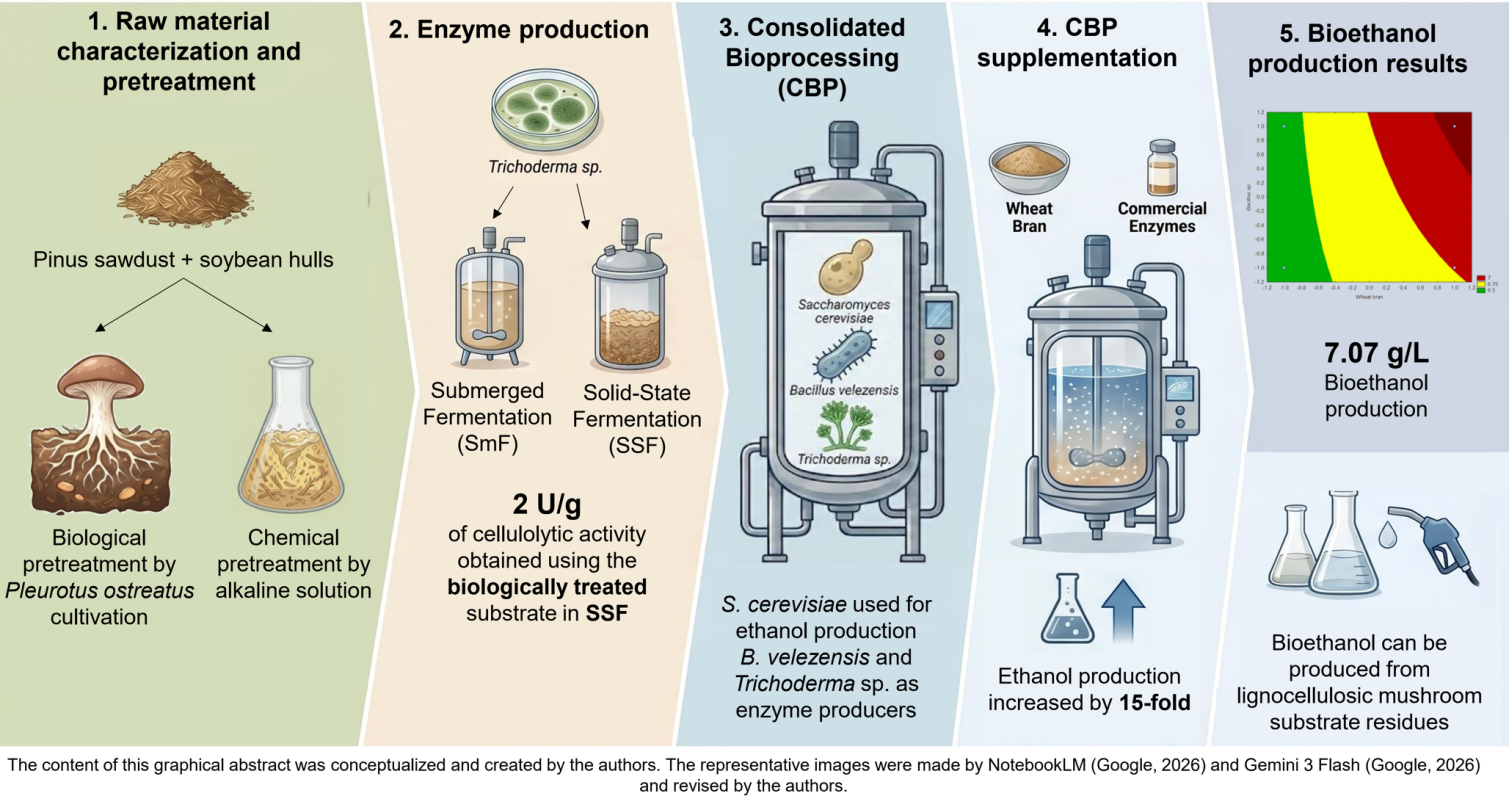

## Introduction

Biomass is a renewable and sustainable energy source derived from the utilization of organic matter of plant or animal origin. It can be converted into biofuels through physicochemical and microbiological processes, offering an alternative to fossil fuels, which are limited and environmentally harmful (Periyasamy et al. 2023). According to Bhatia et al. (2020), lignocellulose is the most abundant, inexpensive, and renewable raw material, representing a promising option for reducing dependence on fossil energy sources. However, the use of lignocellulosic biomass for biofuel production presents challenges due to the recalcitrant nature of its structure, which requires the application of pretreatment methods to enhance its accessibility and facilitate the production of value-added compounds (Ashokkumar et al. 2022).

Given the global context and Brazil’s vast potential for generating renewable resources, it is essential that companies, academic institutions, and research centers invest efforts in the development and improvement of biotechnological processes aimed at the efficient conversion of biomass into biofuels, in order to meet the growing demand for sustainable energy sources (Lorenzi and Andrade 2019; IPCC 2023). In particular, despite its abundance, lignocellulosic biomass remains underutilized, as its conversion requires considerable effort due to its recalcitrant and heterogeneous nature, making the process more complex and technically challenging (Ashokkumar et al. 2022; Devi et al. 2023).

The production of bioethanol from lignocellulosic biomass, known as second-generation (2G) ethanol, is a biotechnological process with significant potential, as demonstrated by numerous studies in the field (Antunes et al. 2022; Shankar et al. 2024). Among the main strategies for 2G bioethanol production are Separate Hydrolysis and Fermentation (SHF), Simultaneous Saccharification and Fermentation (SSF), and Consolidated Bioprocessing (CBP) (Liu et al. 2019). CBP stands out as both an innovative and challenging approach within the context of lignocellulosic biomass conversion. Unlike conventional processes that separate enzyme production, hydrolysis, and fermentation into distinct stages, CBP integrates all these steps into a single system. By eliminating the need for exogenous enzyme addition and reducing the number of unit operations, CBP enhances energy efficiency, lowers operational costs, and minimizes waste generation (Lynd et al. 2005). Thus, it emerges as a promising alternative for improving the economic feasibility of second-generation biofuel production.

For an effective CBP using lignocellulosic biomass, it is preferable to employ substrates with lower recalcitrance to facilitate the action of microorganisms involved in the production of reducing sugars. To achieve this, various pretreatment methods can be applied. The main objective of pretreatment is to initiate the exposure of cellulose fibers by partially or fully solubilizing the lignin and hemicellulose layers, thereby reducing the structural recalcitrance of the biomass. (Saratale et al. 2023; Sriariyanun et al. 2024).

The selection of an appropriate pretreatment method must be carried out carefully, as this step may lead to the formation of by-products and compounds that can inhibit microbial activity (Liu et al. 2019). Biological pretreatments, using microorganisms, enzymes, or other biological agents to degrade the biomass, offer advantages such as lower energy consumption compared to chemical and physical methods, reduced wastewater generation, and a lower formation of inhibitory compounds (Cheng and Whang 2022).

Mushrooms, macroscopic fungal organisms cultivated for food and medicinal purposes, can contribute to improving the bioethanol production process from lignocellulosic biomass (Devi et al. 2022). Their ability to decompose such materials relies on the secretion of lignocellulolytic enzymes, such as cellulases and hemicellulases, which break down complex polymers into simple sugars that the fungi can use as an energy source (Buswell et al. 1996; Saubenova et al. 2023).

It is estimated that for every kilogram of cultivated mushrooms, approximately 3 to 5 kg of residual substrate are generated (Zisopoulos et al. 2016; Kumla et al. 2020). After mushroom harvesting, this residual substrate retains characteristics that make it suitable for bioethanol production, namely, a significant content of cellulose and hemicellulose, while also providing nutrients for fermentation, unlike many other lignocellulosic feedstocks (Chen et al. 2022). A key advantage of using this residual substrate as a raw material is that the prior fungal cultivation promotes partial degradation of the biomass cell wall components, thereby reducing its recalcitrance.

Thus, this study aimed to implement a consolidated bioprocess (CBP) for bioethanol production using residues from shimeji mushroom (*Pleurotus ostreatus*) cultivation, composed of *Pinus elliottii* sawdust and soybean hulls (*Glycine max*). The effects of biological pretreatment (shimeji mushroom cultivation) and alkaline pretreatment (using sodium hydroxide) were evaluated regarding their influence on enzyme production by *Trichoderma* sp. using the residues as substrate. Subsequently, CBP was assessed for ethanol production employing *Trichoderma* sp. and *Saccharomyces cerevisiae*. The effects of wheat bran supplementation, commercial enzyme addition, and co-culture with *Bacillus velezensis* were also investigated.

## Materials and methods

### Raw material characterization

The biomass used consisted of a combination of *Pinus elliottii* sawdust and soybean hulls (*Glycine max*), with a composition of 80% and 20%, respectively. Both the raw material and the spent mushroom substrate (SMS), obtained after the cultivation of oyster mushroom (*Pleurotus ostreatus*), were directly supplied by a local producer. The raw material corresponds to the substrate before mushroom cultivation. Although the mushroom cultivation was performed by the local producer, it was considered a biological pretreatment of the lignocellulosic biomass. The cultivation conditions of the mushroom used by the local producer are described in the section “Pretreatments”.

For the investigation of the proximate composition of the lignocellulosic biomass, samples of the substrate before and after the mushroom cultivation were analyzed for moisture, ash content, protein, lipids, total fiber, neutral detergent fiber (NDF), acid detergent fiber (ADF), and lignin. The analytical procedures followed the Sindirações methodology (Sindirações 2017). Based on the NDF, ADF, and lignin analyses, cellulose and hemicellulose contents were determined according to the Van Soest and Wine method (1967), where cellulose content was calculated as the difference between ADF and lignin content, and hemicellulose content was obtained by the difference between NDF and ADF.

### Pretreatments

The lignocellulosic substrate was subjected to biological and alkaline treatments individually and sequentially according to a 2² experimental design, as shown in Table 1, where (−) indicates the absence of treatment and (+) indicates the application of treatment. In test D, the biological treatment was performed first, followed by the alkaline treatment. All experiments were conducted in triplicate.

**Table 1:** 2² Experimental design conducted for the evaluation of pretreatments

Test	Biological pretreatment	Alkaline pretreatment
A	-	-
B	+	-
C	-	+
D	+	+

The biological pretreatment consisted in the cultivation of the oyster mushroom by the local producer. The cultivation process involved temperature control at 22 °C, with variations ranging from 10 °C to 26 °C, and relative humidity maintained at 96%, ranging from 85% to 99%, over approximately 60 days from the beginning of cultivation to the harvest of fruiting bodies.

The alkaline pretreatment was performed using a biomass loading of 10% (w/v) in a 4% (w/v) sodium hydroxide solution. The treatment was conducted in an autoclave at 110 °C for 30 min. After the process, the solid residues were filtered and rinsed under running water at least three times or until the wash water reached a neutral pH.

Following the pretreatments, the substrates were dried in an oven at constant temperature until a constant weight was achieved, with a minimum drying time of 24 h. The materials were then stored in a dry, dark place until further use.

The effect of the pretreatments on the biomass structure was evaluated by identifying surface functional groups using Attenuated Total Reflectance Fourier Transform Infrared Spectroscopy (ATR-FTIR, Cary 630, Agilent Technologies, USA) in the range of 4000 to 650 cm⁻¹. The mushroom cultivation substrate was analyzed before and after the pretreatments.

In order to evaluate the effect of pretreatments for consolidated bioprocessing (CBP), the residues were used as substrates for solid-state fermentation (SSF) and submerged fermentation (SmF) with *Trichoderma* sp. (Brazilian SisGen registration number A616847) to produce cellulolytic enzymes. Assessing the fungus’s ability to produce cellulolytic enzymes from the residues is essential for the success of CBP. The fermentation method that resulted in the highest enzymatic activity was selected for the subsequent stages of the study. The cellulolytic activities of the produced extracts were quantified as described in Sect. 2.4.1.

### Submerged fermentation (SmF)

Submerged fermentation was carried out in 250 mL Erlenmeyer flasks containing 5% (w/v) of dried substrate, supplemented with 3.0 g/L of (NH₄)₂SO₄, 2.0 g/L of yeast extract, 1.5 g/L of anhydrous glucose, and 10 mL/L of a trace element solution (5 mg/L FeSO₄·7 H₂O; 1.6 mg/L MnSO₄·7 H₂O; 1.4 mg/L ZnSO₄·7 H₂O; and 2 mg/L CoCl₂) (He et al. 2021; Tunio et al. 2024), in a final volume of 100 mL. The medium was sterilized in an autoclave at 120 °C for 15 min. After cooling, inoculation was performed using two 20 mm mycelial discs taken directly from Petri dishes containing PDA medium and active *Trichoderma* sp. cultures. The flasks were incubated in a refrigerated shaker (Oxylab) at 150 rpm and 30 °C for 15 days. Samples were collected periodically and centrifuged at 2500 rpm for 10 min, and the enzymatic activity was determined in the supernatant.

### Solid-state fermentation (SSF)

Solid-state fermentation was conducted in 250 mL beakers containing 5 g of dried substrate with moisture content adjusted to 80%. Moisture adjustment was performed using Mandel’s nutrient solution, composed of: 1.9 g/L (NH₄)₂SO₄; 2.0 g/L KH₂PO₄; 0.3 g/L MgSO₄·7 H₂O; 0.3 g/L CaCl₂; 0.75 g/L peptone; 2 mL/L of 1% Tween 80 solution; 0.63 g/L urea; and 1 mL/L of trace element solution (5 mg/L FeSO₄·7 H₂O; 1.6 mg/L MnSO₄·7 H₂O; 1.4 mg/L ZnSO₄·7 H₂O; and 2 mg/L CoCl₂) (Grujić et al. 2015; Ariff et al. 2019).

The pH of the culture media was adjusted to 4.5 using a 1 mol/L HCl solution. The containers were then covered with acrylic fiber and sterilized in an autoclave at 120 °C for 15 min. After sterilization, 2 mL of a spore suspension containing 10⁶ spores/mL was added to each vessel. The experiments were incubated at 30 °C for 5 days, and samples were collected periodically to determine enzymatic activities.

For enzyme extraction from the solid fermentation medium, 1 g of solid sample was mixed with 10 mL of 0.2 mol/L sodium phosphate buffer (pH 5.5), and the mixture was kept under agitation in a refrigerated water bath at 30 °C for 60 min. After this period, the samples were centrifuged and the supernatant was collected as the crude enzymatic extract.

### Consolidated bioprocessing for ethanol production

The consolidated bioprocessing (CBP) strategy adopted in this study was designed to integrate enzymatic production, substrate hydrolysis, and ethanol fermentation within a sequential and partially overlapping system. Initially, a solid-state fermentation step was employed to promote the production of lignocellulolytic enzymes, followed by a pre-hydrolysis stage to increase the availability of fermentable sugars. Subsequently, alcoholic fermentation was carried out, allowing the simultaneous progression of hydrolysis and fermentation reactions. Based on this strategy, the experimental procedure was carried out as follows.

Accordingly, SSF with *Trichoderma* sp. was conducted for five days under the conditions described in Sect. 2.2.2. After this period, 0.2 mol/L sodium phosphate buffer (pH 5.5) was added, resulting in a reaction medium containing 5% (w/v) solids (based on the initial dry weight of the biomass) in a total volume of 50 mL in 125 mL Erlenmeyer flasks. The flasks were incubated at 50 °C under agitation for 6 h (Sandri et al. 2023).

After pre-hydrolysis, the samples were cooled to room temperature and supplemented with 10% inoculum of *Saccharomyces cerevisiae* CAT-1 (obtained from the University of São Paulo, Ribeirão Preto campus) with an optical density between 0.8 and 1.0 (Rempel et al. 2019). Alcoholic fermentation was then carried out in 125 mL Erlenmeyer flasks with a reaction volume of 50 mL. The flasks were incubated under static conditions at 30 °C for 36 h. At the end of the fermentation process, samples were collected for ethanol quantification, as described in Sect. 2.4.2.

During the CBP process, the following variables were evaluated: medium composition through the addition of wheat bran to the mushroom cultivation residues, supplementation with commercial enzymes, and co-cultivation of *Saccharomyces cerevisiae* CAT-1 with *Bacillus velezensis* UPF-B2 (Brazilian SisGen registration number A9CE908; 16 S rDNA sequences and gyrB genes deposited in NCBI under the numbers OQ570975 and OQ596529, respectively as described by Devos et al. (2024) during alcoholic fermentation. The addition of wheat bran was assessed as a more accessible carbon source aimed at enhancing fungal enzyme production (Lodha et al. 2020), at levels (Devos et al. 2024)of 0 and 10% (w/w) added to the substrate. The use of commercial enzymes during the pre-hydrolysis step was investigated to intensify the conversion of polysaccharides into fermentable sugars (Luo et al. 2024). For this, 5 mL of a commercial enzymatic extract containing cellulases and β-glucosidases (SAE0020, Novonesis) as well as hemicellulases (H2125, Sigma-Aldrich) was added at a 1:10 dilution. The flasks were incubated at 50 °C for 6 h. The co-inoculation of *Bacillus velezensis* UPF-B2 along with *Saccharomyces cerevisiae* was also evaluated during alcoholic fermentation at levels of 0 and 10% (v/v) at the beginning of fermentation, due to the well-documented cellulolytic activity of the bacterium (Zeng et al. 2021), aiming to increase the availability of enzymes capable of degrading lignocellulose. All these parameters were assessed using a 2³ experimental design, as shown in Table 2. At the end of the fermentation process, samples were collected for ethanol quantification, as described in Sect. 2.4.2. All experiments were conducted in duplicate.

**Table 2:** 2³ Experimental design for CBP evaluation using mushroom cultivation residues for ethanol production

Test	Wheat bran (% w/v)	Commercial Enzymes (% v/v)	Co-culture with B. velezensis (% v/v)
1	−1 (0)	−1 (0)	−1 (0)
2	+ 1 (10)	−1 (0)	−1 (0)
3	−1 (0)	+ 1 (10)	−1 (0)
4	+ 1 (10)	+ 1 (10)	−1 (0)
5	−1 (0)	−1 (0)	+ 1 (10)
6	+ 1 (10)	−1 (0)	+ 1 (10)
7	−1 (0)	+ 1 (10)	+ 1 (10)
8	+ 1 (10)	+ 1 (10)	+ 1 (10)

### Analytical methods

#### Determination of cellulolytic activity

Enzymatic activity was determined using carboxymethylcellulose (CMC) as the standard substrate. Cellulolytic activity was measured by mixing 1 mL of the crude enzymatic extract obtained from the fermentations with 1 mL of 1% (w/v) CMC solution prepared in 0.2 mol/L sodium phosphate buffer (pH 5.5), followed by incubation at 50 °C for 30 min. After incubation, the amount of reducing sugars released was quantified using the 3,5-dinitrosalicylic acid (DNS) method (Miller 1959). Control reactions were performed using the 1% CMC solution and heat-denatured enzyme extract. Enzyme denaturation was achieved by boiling 1 mL of the supernatant for 10 min. The control values were subtracted from the corresponding enzymatic reaction readings to discount the reducing sugars already present in the extract.

Enzymatic activity was expressed in units (U), with one unit defined as the amount of enzyme capable of releasing 1 µmol of glucose equivalents per minute under the assay conditions.

#### Determination of ethanol concentration

To determine the concentration of ethanol produced, fermentation samples were distilled using a bench-scale microdistiller (Tecnal, model TE-012). The recovered ethanol was analyzed using the acidified potassium dichromate method at 600 nm (Salik and Povh 1993) (Salik and Povh 1993).

#### Calculation of theoretical ethanol yield

The theoretical ethanol yield from lignocellulosic biomass is based on the conversion of structural carbohydrates (cellulose and/or hemicellulose) into fermentable sugars, which are subsequently metabolized by fermentative microorganisms to produce ethanol. The estimation of the theoretical yield can be performed based on Eq. (1).

1 $$\:Y_{theoretical}=\frac{{m\:etOH}_{produced}}{{m\:etOH}_{theoretical\:from\:biomass}}\times\:100$$

where *Y*_*theoretical*_ is the theoretical ethanol yield (%), *m etOH*_*produced*_ represents the mass of ethanol produced during fermentation (g), and *m etOH*_*theoretical from biomass*_ corresponds to the mass of lignocellulosic biomass theoretically converted to ethanol. This value reflects the maximum possible ethanol yield assuming complete stoichiometric conversion of structural carbohydrates (cellulose) into glucose and subsequently into ethanol (g).

### Statistical analysis

Analysis of variance (ANOVA) was used to statistically analyze the experimental designs. Mean comparisons were conducted using Tukey’s test, with a significance level set at *p* ≤ 0.05. Statistical analysis was performed using Statistic 7.0 software.

## Results

### Characterization of the raw material

Table 3 presents the proximate composition of the lignocellulosic residues before and after mushroom cultivation. A slight increase in protein content was observed in the biomass after undergoing the biological pretreatment.

**Table 3:** Proximate composition (wet basis) of the lignocellulosic biomass (Pinus sawdust and soybean hulls) before and after cultivation of the oyster mushroom

Raw material	Moisture (%)	Protein (%)	Lipids (%)	Ash (%)	Crude Fiber (%)	NFD (%)	ADF (%)
Pre-cultivation	61,07	1,89	0,10	2,24	21,30	31,35	25,26
Post-cultivation	60,04	2,24	0,22	1,73	26,03	35,90	33,75

Based on the proximate composition and lignin quantification results, the cellulose and hemicellulose contents of the samples were estimated, as shown in Table 4. The results indicate that, in both samples, the main components are cellulose and lignin, which is consistent with the typical profile of lignocellulosic biomass.

**Table 4:** Composition of structural carbohydrates (on a dry basis) of lignocellulosic biomass (pine sawdust and soybean hulls) before and after cultivation of the oyster mushroom

Carbohydrate	Pre-cultivation	Post-cultivation
Cellulose (FDA-LIG)	31,54%	45,80%
Hemicellulose (FDN-FDA)	15,64%	5,52%
Lignin (LIG)	33,34%	40,98%

### Evaluation of pretreatments

**Fig. 1:**
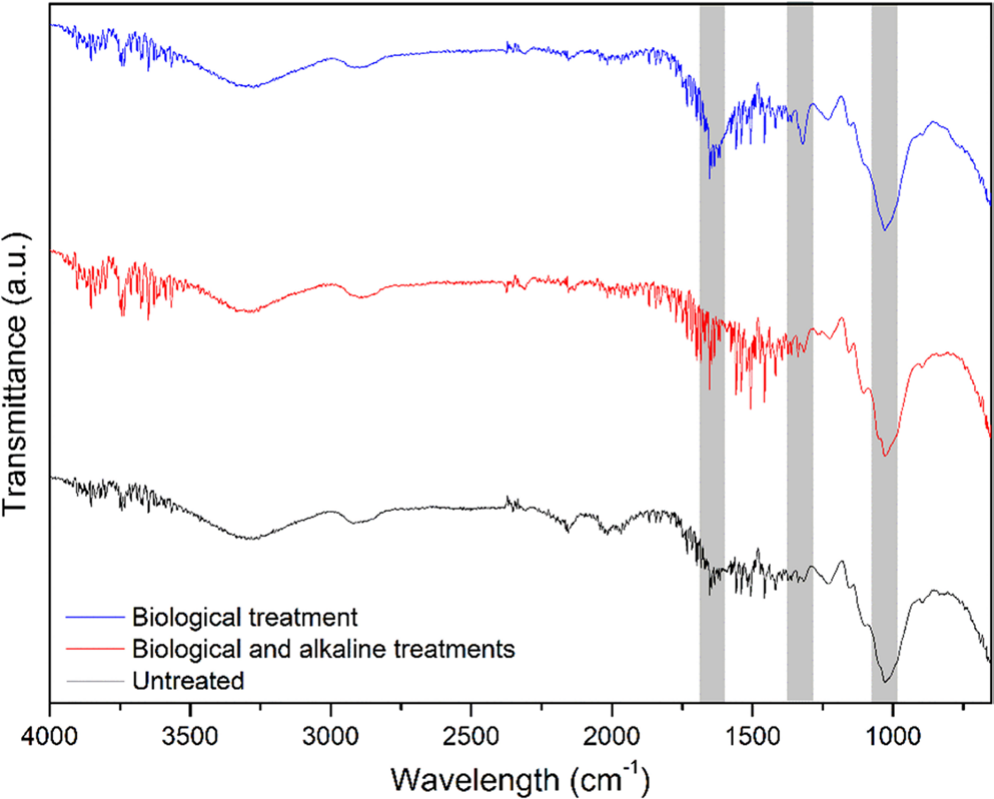
ATR-FTIR of the raw lignocellulosic biomass, after biological pretreatment, and after biological and alkaline pretreatment combined

In order to increase the availability of carbohydrates in the lignocellulosic biomass for microorganisms during CBP, different pretreatments were performed: biological pretreatment through the cultivation of the oyster mushroom, alkaline pretreatment using sodium hydroxide, and a combined biological–alkaline pretreatment. The analysis of the FTIR spectra (Fig. 1) confirmed structural changes in the biomass after the pretreatments.

**Fig. 2:**
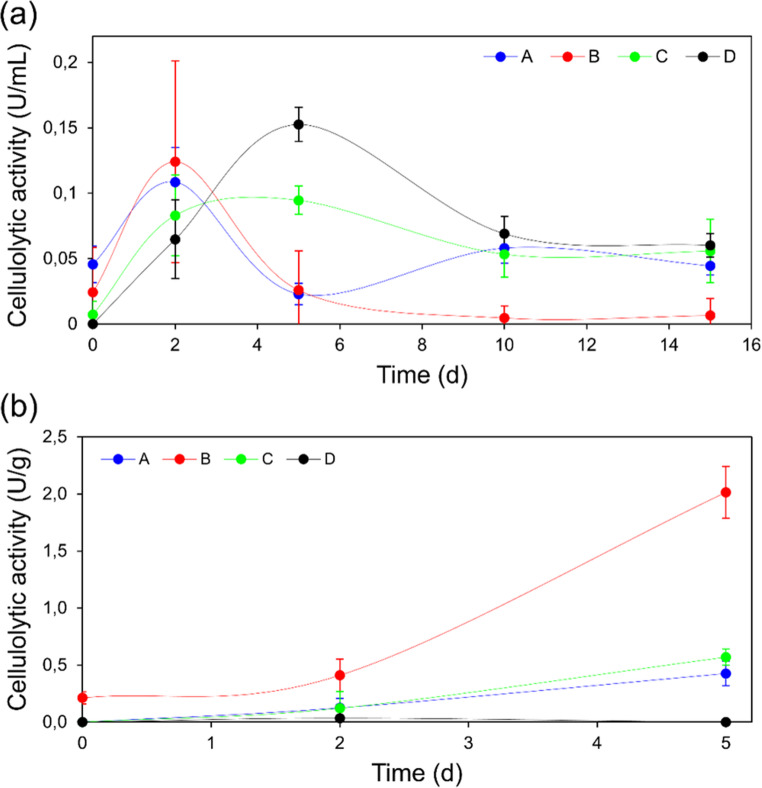
Cellulolytic enzyme activity produced by (**a**) SmF and (**b**) SSF of *Trichoderma* sp. A: untreated substrate; B: substrate with biological pretreatment; C: substrate with alkaline pretreatment; D: substrate with combined biological and alkaline pretreatment

To evaluate the efficiency of the pretreatments, the samples were used as substrates in submerged (SmF) and solid-state (SSF) fermentation by *Trichoderma* sp., and the activity of the cellulolytic enzymes produced by the fungus was determined (Fig. 2). Since this is a CBP process, the ability of the microorganisms to produce enzymes is essential for the success of the process; therefore, the pretreatment must facilitate this enzymatic production.

The analysis of variance (ANOVA) results for enzymatic activity obtained on the 5th day of fermentation (Tables 5 and 6) reveal significant effects of the evaluated pretreatments under both submerged fermentation (SmF) and solid-state fermentation (SSF) conditions.

**Table 5:** ANOVA of enzymatic activity results obtained on the 5th day of experiments from the 2² factorial design for submerged fermentation of *Trichoderma* sp

FACTOR	SS	dF	MS	F	*p*
Alkaline Pretreatment (1)	0,0392	1	0,0392	126,513	0,000000
Biological Pretreatment (2)	0,0037	1	0,0037	12,003	0,004677
1 by 2	0,0030	1	0,0030	9,763	0,008778
Error	0,0037	12	0,0003		
Total SS	0,0497	15			

**Table 6:** ANOVA of enzymatic activity results obtained on the 5th day of the experiments from the 2² factorial experimental design for solid-state fermentation of *Trichoderma* sp

FACTOR	SS	dF	MS	F	*p*
Alkaline Pretreatment (1)	5,1049	1	5,1049	289,415	0,000000
Biological Pretreatment (2)	0,2221	1	0,2221	12,591	0,004008
1 by 2	3,1301	1	3,1301	177,456	0,000000
Error	0,2116	12	0,0176		
Total SS	8,6688	15			

In SmF (Table 5), the alkaline pretreatment was the most significant variable, followed by the biological pretreatment and the interaction between them. These results suggest that although both individual treatments contributed to improved enzymatic activity, the chemical disruption of lignocellulosic structure through alkaline pretreatment was more effective in enhancing enzyme secretion in submerged conditions. 

Under SSF (Table 6), the differences were even more striking. The alkaline pretreatment accounted for the most significant portion of the total variance, with the biological pretreatment also showing a significant though smaller effect. 

The standardized estimated effects (Table 7) provide additional insight into the direction and magnitude of each factor’s contribution. In SmF, all factors had positive effects on enzymatic activity, with the alkaline pretreatment showing the strongest influence, followed by the biological pretreatment and their interaction. Conversely, in SSF, the alkaline pretreatment showed a negative standardized effect.

**Table 7:** Standardized values of the estimated effects of the alkaline and biological pretreatments of the mushroom substrate in the enzyme production by SmF and SSF

FATOR	SmF	SSF
Alkaline Pretreatment (1)	11,247	−17,012
Biological Pretreatment (2)	3,464	3,548
1 by 2	3,124	−13,321

### Consolidated bioprocess for ethanol production

**Fig. 3:**
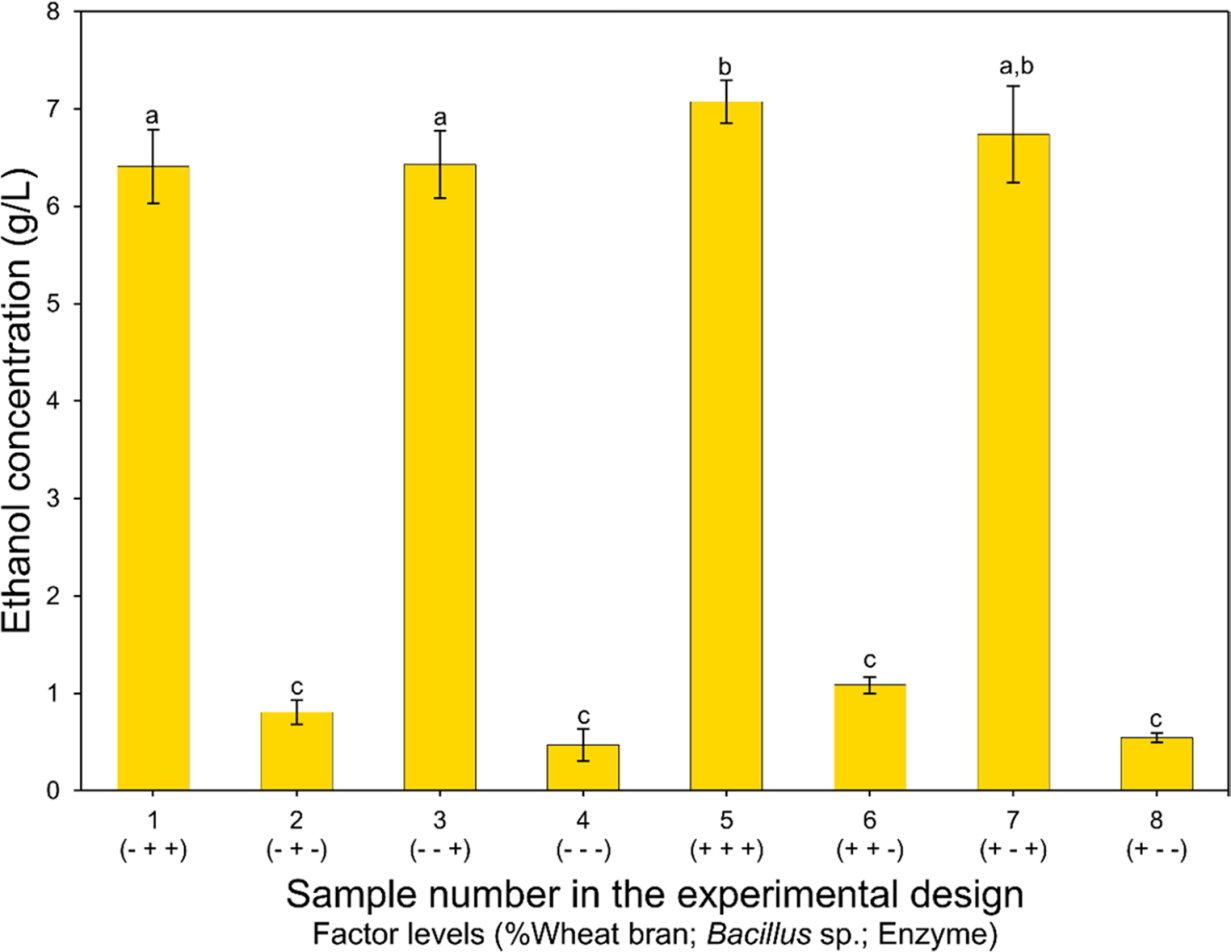
Ethanol concentration obtained in the 2^3^ factorial design experiments, evaluating the effects of wheat bran, commercial enzymes, and *Bacillus velezensis* inoculum on the consolidated bioprocess (CBP). Bars labeled with the same letter did not show significant differences according to Tukey’s test (*p* < 0.05)

Based on the results of the pretreatments, the study on the consolidated bioprocessing (CBP) for ethanol production was conducted using only the biomass after mushroom cultivation. Figure 3 shows the results of the 2^3^ factorial experimental design performed to evaluate the addition of wheat bran to the lignocellulosic biomass, the addition of commercial enzymes during the pre-hydrolysis step, and co-culture with *Bacillus velezensis*. Ethanol concentrations ranged from 0.470 ± 0.167 g/L (sample 4, without addition of any of the evaluated components) up to a maximum of 7.075 ± 0.218 g/L (sample 5, with the addition of all evaluated components). Experiments 1, 3, 5, and 7, which received the addition of commercial enzyme, exhibited ethanol concentrations considerably higher than the others, all above 6 g/L.

The results obtained for samples 2, 4, 6, and 8 indicate considerably low ethanol concentrations, below 1.1 g/L. When analyzing the ethanol yield in comparison to the theoretical maximum yield calculated based on the amount of cellulose present in the biomass, it was observed that the highest yield achieved, when no enzyme was added to the process, was only 8.94%. In contrast, when enzymes were supplemented, the yield reached 57.0%. These data highlight the low efficiency of ethanol production without enzyme supplementation, suggesting that the enzymes produced by *Trichoderma* sp. and *Bacillus velezensis* during fermentation were insufficient for effective conversion.

**Fig. 4:**
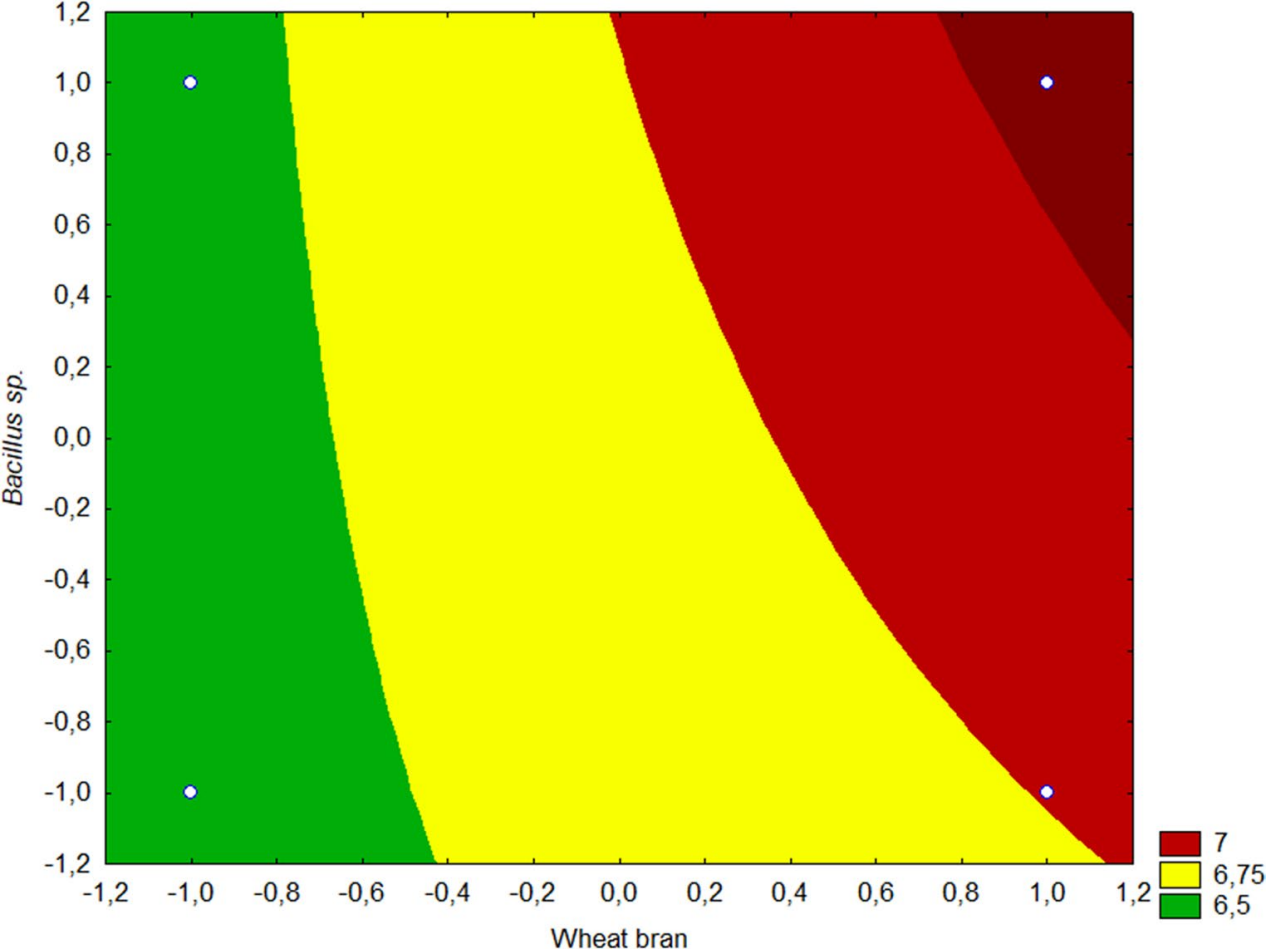
Contour plots of ethanol concentration as a function of wheat bran addition and*Bacillus velezensis* inoculum (the concentration of commercial enzymes was fixed at the 10% v/v)

The results of the experimental design for ethanol production by CBP were subjected to analysis of variance (ANOVA) (Table 8). The coefficient of determination (R² = 0.99) and the F-test validated the model (*p* < 0.05), allowing the generation of the contour plot shown in Fig. 4.

**Table 8:** ANOVA of ethanol concentration results obtained from the 2^3^ factorial experimental design for CBP evaluation

FATOR	SS	dF	MS	F	*p*
Wheat bran (1)	0,8832	1	0,8832	12,063	0,001888
Commercial enzyme (2)	281,8656	1	281,8656	3849,643	0,000000
*Bacillus velezensis* (3)	0,7117	1	0,7117	9,721	0,004542
1 by 2	0,1935	1	0,1935	2,642	0,116591
1 by 3	0,1573	1	0,1573	2,148	0,155211
2 by 3	0,1573	1	0,1573	2,148	0,155211
Error	1,8305	25	0,0732		
Total SS	285,7990	31

As expected, the added enzyme has a highly significant impact on ethanol concentration. Furthermore, the addition of wheat bran and *Bacillus velezensis* inoculum, when evaluated at a 5% significance level, shows p-values indicating that both factors also statistically significantly affected ethanol production. It can be observed that the highest ethanol production occurs in the region corresponding to elevated levels of all factors, indicating more favorable conditions for the bioprocess, specifically at 10% wheat bran supplementation during enzyme production and 10% bacterial inoculum during alcoholic fermentation.

## Discussion

### Characterization of the raw material

The proximate composition of the raw material shows an increase in protein levels in the mushroom substrate after mushroom cultivation, which can be attributed to fungal metabolism and may benefit subsequent fermentation processes by providing a more nutrient-rich medium for microbial growth and activity (Khalil et al. 2011). The structural carbohydrates composition shows the predominance of cellulose, which was expected since it is the primary constituent of the plant cell wall, while the high proportion of lignin is attributed to the woody nature of the material (Nanda et al. 2016).

The most significant change caused by mushroom cultivation was observed in the hemicellulose content, which decreased by over 10%. Reductions in cellulose and hemicellulose contents were expected, considering the fungus’s metabolic preference for these carbon sources (Öztürk and Atila 2021). The variation in composition observed in our pretreatment is similar to that reported by Klausen et al. (2023). In their study, using a hardwood-based substrate containing approximately 36% cellulose, 28% hemicellulose, and 19% lignin, after cultivation of two Pleurotus ostreatus strains, the final substrate composition was 37.5–38.3% cellulose, 20.4–22.1% hemicellulose, and 16–18.4% lignin.

Although the biological treatment consumes cellulose/glucose, which are compounds of interest for fermentation, the enzymatic activity of these organisms can cause significant alterations in the cell wall structure of the biomass. Enzymes such as laccases and peroxidases promote lignin degradation, while cellulases reduce the crystallinity index of cellulose (Rajavat et al. 2020; Sardar et al. 2024). These modifications can make the remaining biomass more susceptible to enzymatic hydrolysis.

### Evaluation of pretreatments

The FTIR shows bands at the wavelength of 1320 cm⁻¹ which corresponds to vibrations related to C–H groups found in cellulose and other polysaccharides. Increases in this peak are associated with the fungal effect, which preferentially consumes amorphous cellulose, resulting in a higher proportion of crystalline cellulose in the final material (Apaydın Varol and Mutlu 2023; Akcay et al. 2023).

Given that lignin is the predominat aromatic component in biomass, the spectral region from 1500 to 1600 cm⁻¹, probably associated with C = C stretching vibrations from lignin aromatic rings, showed a decrease in intensity for the substrate subjected to alkaline pretreatment, indicating lignin removal (Haldar and Purkait 2022; Wang et al. 2025) (Haldar & Purkait, 2020; Wang et al. 2025). In contrast, the substrate treated biologically exhibited an increase in the intensity of these bands, possibly related to a higher lignin content in the pretreated substrate and the presence of metabolic by-products from lignin degradation, which is in accordance with the results shown in Table 4 for the composition of structural carbohydrates of the lignocellulosic biomass before and after cultivation of the oyster mushroom.

In the SmF of *Trichoderma* sp., the substrate subjected to both biological and alkaline pretreatments proved to be the most favorable for enzymatic production, reaching the highest cellulolytic activity (0.152 ± 0.012 U/mL) on the fifth day of cultivation. SSF more closely resembles the natural growth conditions of filamentous fungi (Singhania et al. 2021) and resulted in higher enzymatic yields, reaching 2.04 ± 0.17 U/g on the fifth day of cultivation. However, in SSF, the most favorable pretreatment for enzymatic production was the biological one alone. The alkaline pretreatment may have led to the release of toxic by-products, such as phenolic acids, which inhibit microbial metabolism and negatively affect enzyme production (Qin et al. 2016; Xie et al. 2018). Similarly, Legodi et al. (2023) concluded that alkaline pretreatment did not improve cellulase production in SSF using pseudostem banana biomass and *Trichoderma longibrachiatum*. Cellulolytic activity values decreased from 70 FPU/gds with the untreated biomass to 21 FPU/gds when the biomass was pretreated with 3% NaOH.

Based on the SmF and SSF results, the SSF lead to a higher enzymatic activity and was only positively affected by the biological pretreatment. Therefore, for the next steps, SSF was selected for a pre-production of cellulolytic enzymes using the raw material treated only by the mushroom cultivation.

### Consolidated bioprocess for ethanol production

It is evident that the presence of the commercial enzyme played a crucial role in enhancing the efficiency of the fermentation process, likely by intensifying the hydrolysis of substrate polymers and making a greater amount of monomeric sugars available for fermentation. Lignocellulosic biomass is composed of cellulose and hemicellulose tightly bound within a lignin matrix, and its efficient deconstruction requires a broad spectrum of hydrolytic enzymes, particularly endoglucanases, exoglucanases, β-glucosidases, and hemicellulases (Lynd et al. 2005). While microorganisms such as *Trichoderma sp.* and *Bacillus velezensis* are capable of producing some of these enzymes, the levels and diversity of enzyme activity under CBP conditions may not be sufficient for complete saccharification. Similar results were reported by Sandri et al. (2023), who employed a simultaneous saccharification and co-fermentation (SSCF) strategy using pretreated sugarcane bagasse with *Saccharomyces cerevisiae* and *Kluyveromyces marxianus*, resulting in ethanol production of 6 g/L. Although microorganisms such as *Trichoderma* sp. and *Bacillus velezensis* are capable of producing some of these enzymes, the enzymatic levels and diversity achieved under consolidated bioprocessing conditions may not be sufficient to sustain an adequate rate of saccharification. Consequently, the limited availability of fermentable sugars, rather than the fermentative capacity of the yeast, represents the primary bottleneck of the system in the absence of commercial enzymatic supplementation, highlighting insufficient hydrolysis as the main limiting factor in the evaluated CBP system (Fortuin et al. 2025).

As shown in Fig. 4, the addition of wheat bran to the lignocellulosic substrate positively influenced ethanol production, likely due to its nutritional composition and ease of microbial assimilation. Wheat bran is rich in starch, which can serve as readily available carbon sources for both enzyme-producing and fermentative microorganisms (Carvalho et al. 2008; Tunio et al. 2024). Its presence may have stimulated the metabolic activity of *Trichoderma sp.* during the solid-state fermentation stage, enhancing cellulase production, as well as improved yeast performance during ethanol fermentation. Moreover, the more accessible carbohydrates present in wheat bran can be easily hydrolyzed and fermented by *Saccharomyces cerevisiae*, potentially increasing sugar availability. These effects combined may explain the significant improvement in ethanol yields observed when wheat bran was included in the CBP.

The observed increase in ethanol production with the addition of *Bacillus velezensis* (Fig. 4) may be attributed to the synergistic role this microorganism plays in the consolidated bioprocessing (CBP) system. Several studies have demonstrated that strains of *Bacillus* are capable of producing a range of hydrolytic enzymes, including cellulases and hemicellulases, which aid in the breakdown of complex carbohydrates into fermentable sugars (Zeng et al. 2021). In addition, certain *Bacillus* species exhibit ligninolytic activity, contributing to the depolymerization of lignin and improving the accessibility of cellulose and hemicellulose to enzymatic action (Zhong et al. 2022). This dual enzymatic potential, comprising both cellulolytic and ligninolytic activities, not only enhances the saccharification of lignocellulosic substrates but also reduces the inhibitory effects associated with lignin-derived compounds. As a result, the co-inoculation of *B. velezensis* with *Saccharomyces cerevisiae* likely contributed to the observed improvement in ethanol yield by facilitating more efficient substrate utilization within the CBP system.

## Conclusions

The consolidated bioprocess (CBP) proved to be a promising strategy for bioethanol production from lignocellulosic residues composed of pine sawdust and soybean hulls derived from mushroom cultivation. Biological pretreatment with cultivation of the oyster mushroom, the addition of wheat bran to the pretreated biomass, the supplementation of commercial enzymes during pre-hydrolysis, and the co-culture of the yeast *Saccharomyces cerevisiae* with *Bacillus velezensis* were key factors for the success of the process. Using 10% (w/w) wheat bran, 10% (v/v) of the commercial enzymatic extract containing cellulases, β-glucosidases, and hemicellulases, and 10% (v/v) inoculum of *Bacillus velezensis*, ethanol concentration reached 7.075 ± 0.218 g/L, with a theoretical yield of 57%. Although the need for enzyme supplementation indicates that full enzymatic self‑sufficiency was not achieved, these results do not detract from the relevance of CBP. Instead, they highlight both the current limitations of microbial saccharification under consolidated conditions and the potential of CBP as a long‑term strategy. By integrating enzyme production, hydrolysis, and fermentation into a single process, CBP retains intrinsic advantages in terms of process simplification, reduced unit operations, and potential cost reduction. Continued advances in microbial strain development, enzymatic efficiency, and coculture optimization are therefore expected to further improve CBP performance and support its future application in lignocellulosic bioethanol production. 

## Data Availability

The datasets generated during and/or analyzed during the current study are available from the corresponding author on reasonable request.
